# Effects of virtual reality high heights exposure during beam-walking on physiological stress and cognitive loading

**DOI:** 10.1371/journal.pone.0200306

**Published:** 2018-07-06

**Authors:** Steven M. Peterson, Emily Furuichi, Daniel P. Ferris

**Affiliations:** 1 Department of Biomedical Engineering, University of Michigan, Ann Arbor, Michigan, United States of America; 2 School of Kinesiology, University of Michigan, Ann Arbor, Michigan, United States of America; 3 J. Crayton Pruitt Family Department of Biomedical Engineering, University of Florida, Gainesville, Florida, United States of America; University of Saskatchewan, CANADA

## Abstract

Virtual reality has been increasingly used in research on balance rehabilitation because it provides robust and novel sensory experiences in controlled environments. We studied 19 healthy young subjects performing a balance beam walking task in two virtual reality conditions and with unaltered view (15 minutes each) to determine if virtual reality high heights exposure induced stress. We recorded number of steps off the beam, heart rate, electrodermal activity, response time to an auditory cue, and high-density electroencephalography (EEG). We hypothesized that virtual high heights exposure would increase measures of physiological stress compared to unaltered viewing at low heights. We found that the virtual high height condition increased heart rate variability and heart rate frequency power relative to virtual low heights. Virtual reality use resulted in increased number of step-offs, heart rate, electrodermal activity, and response time compared to the unaltered viewing at low heights condition. Our results indicated that virtual reality decreased dynamic balance performance and increased physical and cognitive loading compared to unaltered viewing at low heights. In virtual reality, we found significant decreases in source-localized EEG peak amplitude relative to unaltered viewing in the anterior cingulate, which is considered important in sensing loss of balance. Our findings indicate that virtual reality provides realistic experiences that can induce physiological stress in humans during dynamic balance tasks, but virtual reality use impairs physical and cognitive performance during balance.

## 1. Introduction

Humans regularly perform activities of daily living and tasks of mobility that require maintenance of dynamic balance. With human aging, balance control can deteriorate [1], leading to falls and other serious consequences [2]. In addition, falls can induce a fear of falling again, potentially leading to a loss of independence [3].

Balance training often reduces the risk of falling [4,5], even more than basic walking tasks [6]. Balance training equipment varies widely from wobble boards [7] to complex balance systems [8]. Because integrating balance training into everyday activities reduces falls [9], virtual reality has been used to motivate users to perform challenging balance tasks in realistic scenarios [10,11]. Dynamic training in virtual reality has improved walking speed for Parkinson’s patients [12] and walking stability for stroke patients [13]. However, many studies project virtual environments onto a screen, which does not move with the user and allows the user to look away [14,15]. Virtual reality presented using a head-mounted display may provide more effective immersion.

To test the realism of a head-mounted display virtual environment during dynamic balance, we exposed subjects to high heights in virtual reality while walking on a balance beam. High heights anxiety is both prevalent [16] and measurably affects dynamic and static stability [17,18]. Human physiological stress levels increase at higher heights and do not noticeably differ across age groups [19]. Immersive virtual reality can provide a cognitive sense of presence where the user feels that they are in a real environment [20,21]. Virtual reality heights exposure is comparable to real-world heights exposure [22], with virtual high heights increasing measures of fear and altering standing posture dynamics [23,24]. In addition, presence during virtual heights exposure can be enhanced by having subjects stand with their feet on a ledge raised only a few centimeters off the ground [25]. Given these results, virtual high heights should alter stress levels during a dynamic locomotor task.

Despite advances in physiological recording methods, stress remains challenging to quantify. Cortisol level is considered one of the best standards for stress detection because cortisol is generated by the hypothalamus-pituitary-adrenal axis directly in response to stress, but measuring cortisol from blood or urine is invasive. Salivary cortisol, while non-invasive, has less fine time resolution than blood cortisol, creating a time lag between stress and cortisol levels [26]. Heart rate variability is affected by both parasympathetic and sympathetic activity, which vary based on stress levels [27]. It has been generally thought that stress induces less heart rate variability [28,29], but results are conflicting and likely depend on the paradigm and stressor used [30]. Electrodermal activity may also indicate stress, as it is affected by sympathetic activity [31]. Electrodermal activity contains a tonic (slow) and phasic (fast) component [32], with increased phasic activity relating to increases in stress [33,34]. Other ways to quantify stress include cognitive task performance [35,36] and EEG activity [30]. Our primary outcome measures of stress were electrodermal activity and heart rate variability because of their direct connections to sympathetic and parasympathetic responses and the ease of recording them during a dynamic balance task.

In addition to stress, we wanted to quantify the physical and cognitive effects of virtual reality use. A head-mounted display moves with the user, which may be advantageous for dynamic balance training compared to screen displays, but immersive virtual reality may induce motion sickness. Motion sickness varies greatly across people and virtual reality setups [37], so it is important to limit and quantify its effects. To estimate cognitive loading, we measured response time to an auditory stimulus [38,39]. We also wanted to know where a potential change in cognitive load was occurring in the brain. For this, we used EEG source localization, which has revealed areas of the brain involved during balance beam walking [40]. This added complexity helped us determine which cognitive centers were most affected by virtual reality use during beam-walking. EEG has been used before to measure cognitive response to stimuli in virtual reality [41].

The purpose of this study was to determine if high height exposure in virtual reality induced stress and if virtual reality use affected physical and cognitive performance during a dynamic balance-beam walking task. Our hypotheses were: 1) subjects’ stress would increase at a high virtual height compared to a low virtual height, as measured by increases in heart rate variability and electrodermal activity, and 2) virtual reality use during beam-walking would increase cognitive load compared to no virtual reality use during the task, as measured by increased response time to an auditory stimulus and decreases in EEG event-related activity peak amplitude. We included a virtual reality low height condition, which matched the beam height of the unaltered view low height condition, for this second comparison. We found that high virtual heights induced stress, and virtual reality use at low heights increased cognitive loading compared to beam-walking without the headset, confirming both hypotheses.

## 2. Materials and methods

### 2.1. Subjects

Human subject research was approved by the University of Michigan Health Sciences and Behavioral Sciences Institutional Review Board (HUM00100932) for the protection of human subjects. All subjects provided written informed consent. Nineteen healthy subjects participated in the study (10 male, age 23±4 years old (mean±SD)). All subjects identified themselves as right hand and right foot dominant. Subjects were screened for any orthopedic, cardiac, or neurological conditions and injuries. Any subjects indicating they experienced acrophobia (fear of heights) were excluded from the study because we wanted all subjects to be able to complete the full experiment.

Prior to the main experiment session, we screened subjects for motion sickness in virtual reality. Subjects stood in place while wearing the headset (Oculus Rift DK2, Oculus VR, Irvine, CA) for 5 minutes. Subjects moved around a virtual environment using body gestures tracked by a Microsoft Kinect V2 (Microsoft, Redmond, WA). We intentionally included this disconnect between real and virtual movements to be more disorienting than the experiment. Subjects were allowed to participate in the main experiment if both the experimenter and subject agreed that the subject did not exhibit any symptoms of motion sickness. Two subjects exhibited symptoms of motion sickness and did not perform the experiment; 19 subjects passed this screening process.

### 2.2. Experiment setup

We tested subjects on a 3.8 cm-wide by 2.5 cm-tall by 3.05 meter-long wooden balance beam, similar to previous studies [42,43]. We attached the beam to a non-skid surface to prevent it from slipping. Subjects walked the entire length of the beam in one direction, referred to as a beam pass. After completing a beam pass, subjects walked over-ground back to their starting position. Subjects walked heel-to-toe with their arms crossed over their chest. We did not make subjects follow a specific gait speed to avoid any effects from attending to this speed. We demonstrated a desired pacing of 0.22 m/s and informed the subject if he or she was walking too fast or too slow. We chose this speed based on previous beam-walking experiments [40,42]. We also instructed subjects to look at their feet while balancing in all 3 conditions.

**Fig 1:**
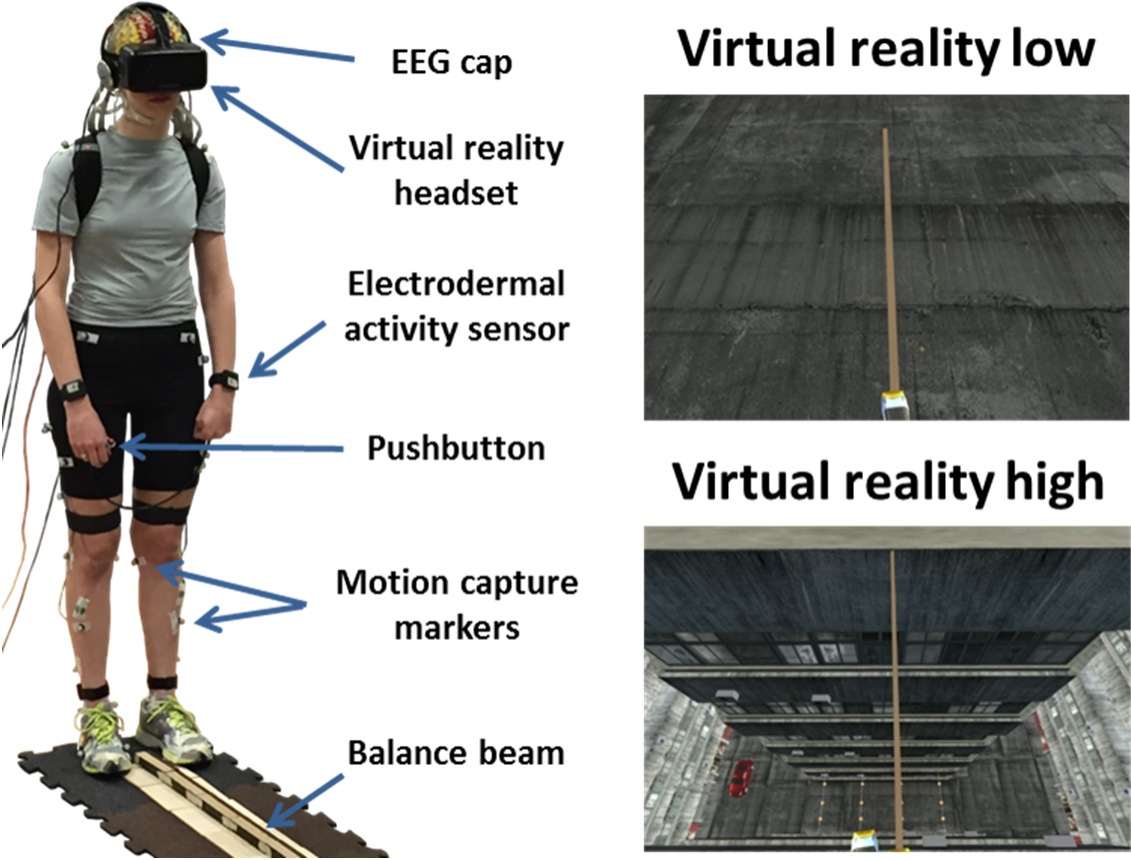
Subject setup and virtual reality views. Typical subject setup is shown (left). All subjects walked on the same physical beam for all three conditions. In both virtual reality conditions, subjects saw a virtual beam that aligned with the physical beam. In virtual reality low, the virtual beam was the same height off the ground as in unaltered view low (top right), while the virtual beam was 15 meters off the ground in virtual reality high (bottom right).

Subjects performed the same physical beam-walking task under 3 viewing conditions: unaltered view low, virtual reality low, and virtual reality high. Unaltered view low involved normal viewing without virtual reality. For virtual reality low and virtual reality high, subjects wore the Oculus virtual reality headset. Subjects viewed themselves 2.5 cm off the ground in virtual reality low, which agreed with the real-world balance-beam height, and 15 meters off the ground in virtual reality high (Fig 1). To enhance the effects in the virtual reality high condition, subjects “fell” 15 meters in the virtual environment when they stepped off the beam. Both virtual reality conditions contained a virtual beam that was aligned with the physical beam. In all 3 conditions, subjects performed the same balance task on the physical beam. Subjects took 10 minute breaks between each condition. We randomized the order of the virtual reality conditions, but all subjects performed the unaltered view low condition second to spread out virtual reality use during the experimental session.

The virtual environment was rendered using Unity 5 software (Unity Technologies, San Francisco, CA) and included a virtual avatar controlled by the Microsoft Kinect. This computer used a NVIDIA Titan X graphics card (NVIDIA, Santa Clara, CA) to avoid slow-downs in the virtual reality presentation. Because humans more reliably perceive heights when they have a body in virtual reality [44], each subject had a virtual avatar. This avatar mimicked the subject’s movements in the virtual environment, using the Kinect tracking with the ‘Kinect v2 Examples with MS-SDK’ Unity package. We did not have the Kinect control the avatar’s arms, hands, and toes because the Kinect could not reliably track them during the experiment. Because the Kinect can only reliably track a user that faces it, subjects made beam passes in one direction and walked over-ground in the other direction. Each condition ended after 15 minutes of forward beam passes. We choose a fixed time instead of a fixed number of passes so that subjects were not encouraged to walk faster.

### 2.3. Performance & physiological measures

While beam-walking, subjects wore several sensors to measure physiological and cognitive activity. To determine gait events, we placed 30 reflective motion capture markers placed on the feet and legs of each subject, sampled at 100 Hz (Vicon, Los Angeles, CA). A wearable device (Empatica E4) was placed on both wrists to record electrodermal activity (Empatica, Milan, Italy). We recorded from both wrists to average out any unreliable activity [45]. Fig 1 shows a representative subject during testing. Subjects also completed surveys after the experiment ended to assess motion sickness in virtual reality (Motion Sickness Assessment Questionnaire [46]) and high heights apprehension (Heights Interpretation Questionnaire [47]).

We analyzed beam-walking performance using motion capture markers at each foot. Marker traces were cleaned in Vicon Nexus and further processed in Visual3D (C-Motion, Germantown, MD). We implemented a similar algorithm as Zeni et al. to find gait events and manually inspected each trial to ensure accuracy [48]. We quantified balance performance by determining the number of times balance was lost divided by the total time spent on the beam. This metric is known as failures per minute and has previously assessed beam-walking performance [42,43]. By including the total time spent on the beam, faster walkers are not rewarded more than slower walkers for making fewer mistakes. In addition, we recorded the number of beam passes for each condition. We also estimated beam-walking speed by calculating the time subjects were on the beam and using the total number of passes as an estimate of distance.

We recorded heart rate via an electrode taped over the sternum. We reduced line noise using the Cleanline EEGLAB plugin and bandpass filtered between 5 and 20 Hz. Kubios HRV software found the R-peaks, which correspond to heartbeats [49]. We manually adjusted incorrectly labelled peaks. We determined heart rate variability as the standard deviation of the heart rate time series. We calculated heart rate frequency power by taking the fast Fourier transform power spectrum of the inter-beat intervals and determining the percent of total power not in the 0.04–0.15 Hz range. Kubios calculated all heart rate and heart rate variability metrics based on guidelines in the field [50].

Electrodermal activity data was processed using Ledalab [51,52], which uses deconvolution to separate the tonic and phasic components of the signal. We ran deconvolution with the default parameters. Electrodermal activity responses were calculated as differences in the deconvoluted phasic signal greater than 0.05μS [53]. We averaged responses across each condition and normalized by condition duration in minutes.

While beam-walking, subjects were instructed to listen for an auditory tone and press a pushbutton upon hearing the tone. We designed this secondary task to be simple because balance performance may worsen if it is too challenging [54,55]. Tones were spaced randomly 7–9 seconds apart, consistent with previous research [56]. We recorded the time subjects took to respond to the tone as an estimator of cognitive load during the beam-walking task. Increased cognitive load during beam-walking would be accompanied by decreased attention to the auditory tone task, resulting in increased response times.

### 2.4. Auxiliary experiment

To determine if any differences found in our measures were caused by simply wearing the headset, we performed an auxiliary experiment on 20 subjects (10 male, age 24±5 years old (mean±SD)). Four subjects participated in both the main and auxiliary experiments, but on separate days. All subjects gave written informed consent, and the protocol was approved by the University of Michigan Institutional Review Board for the protection of human subjects. Subjects performed 4 randomized 5-minute blocks of sitting and standing, both with and without the headset. Subjects were asked to stand and sit up straight while staring at a fixation cross displayed at eye level. We recorded the same electrodermal activity, response time, and heart rate metrics as the main experiment.

### 2.5. Fatigue assessment

Because we were concerned about fatigue, we quantified changes in failures per minute, heart rate, and response time during each condition. We chose these measures because they estimate motor performance, physical exertion, and cognitive loading, each of which can be affected by fatigue. We calculated percent change as the difference between the last and first 3 minutes of each condition, all divided by the first 3 minutes and converted to a percent. We divided the difference by the first 3 minutes because we wanted to see how each measure changed relative to its initial value during the first 3 minutes. If fatigue was present, we would expect to see a large percent change from the first 3 minutes to the last 3 minutes.

### 2.6. EEG data

In addition to response time, we recorded EEG to determine if specific brain areas showed increases in cognitive load during beam-walking. By comparing peak EEG activity following the tone, we can determine changes in electrocortical activity across conditions. Because an increase in cognitive loading during the main task likely results in less focus on the secondary task, we would expect a corresponding decrease in event-related peak amplitude [57,58]. We performed independent components analysis (ICA) to find brain source activity from the channel data [59]. We used ICA because event-related potentials show distinct activity from compact sources in the brain [60]. Unlike response time, EEG with source localization provides insight into cognitive loading differences in specific brain areas. We recorded EEG using a 136-channel BioSemi Active II system (BioSemi, Amsterdam, NL), sampled at 512 Hz. The EEG AD-box and battery were placed in a backpack worn by the subject [61].

EEG data was processed using custom scripts in EEGLAB [62]. We high-pass filtered the data at 1 Hz and removed noisy channels [63,64]. We removed 12±8 bad channels (mean±SD) and interpolated them to maintain a consistent montage across the head. We ran AMICA 15 on the data [65,66], using principal component analysis to reduce down to 100 principal components prior to ICA. This was less than the minimum number of channels remaining following bad channel removal (102 channels), ensuring that the data sent into ICA remained full rank.

After obtaining independent components, we fit the ICA scalp maps to equivalent current dipoles using DIPFIT2 [67]. Independent components with residual variance <15% were retained for further analysis. We manually rejected independent components with non-brain activity, using power spectra and dipole location. We manually rejected 17±4 dipoles and retained 7±3 (mean±SD) cortical dipoles per subject. Brain dipoles were grouped using k-means clustering, using weights of 10, 2, and 1 for dipole location, power spectra, and scalp maps, respectively.

**Fig 2:**
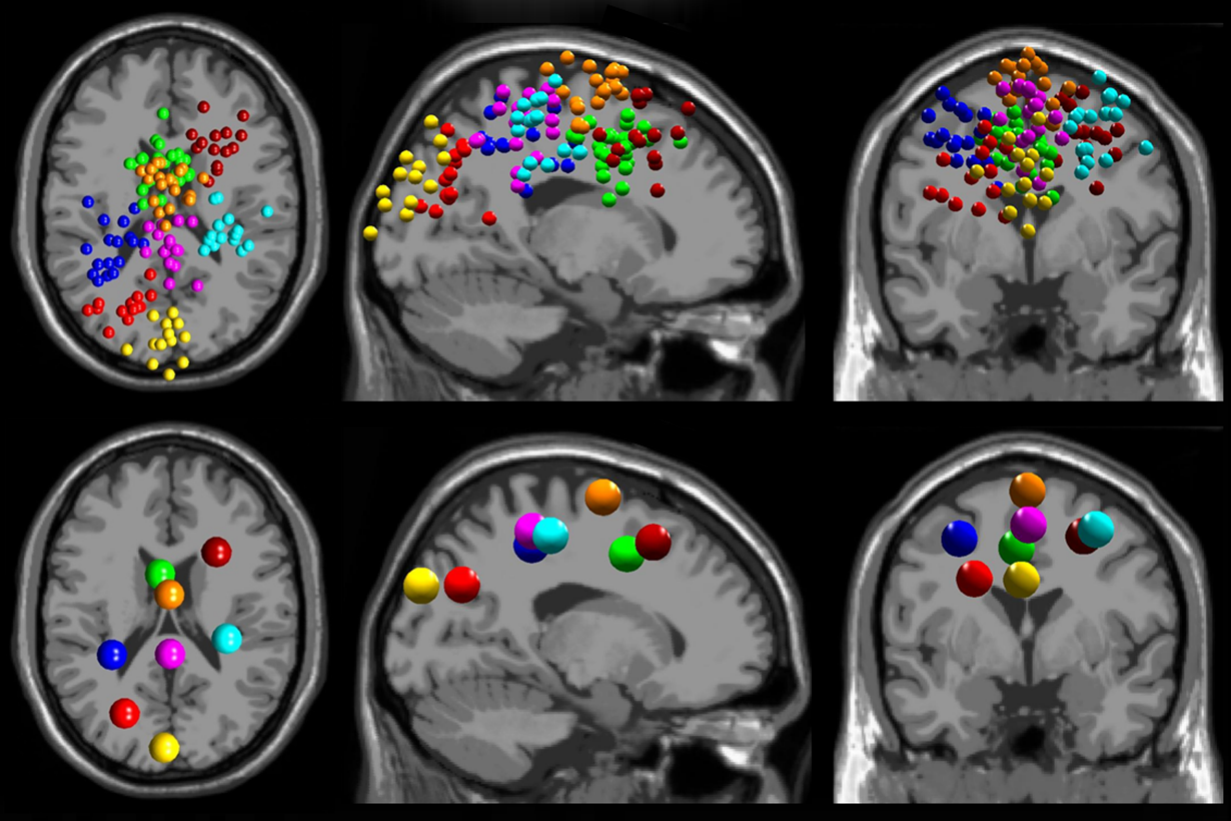
EEG source localization results. EEG source localization results are shown for the 8 cortical clusters found across all subjects (n = 19). Dipole locations (top) and cluster centroids (bottom) are shown in transverse, sagittal, and coronal views (left to right). We found clusters in anterior parietal (purple), left sensorimotor (blue), right frontal (maroon), anterior cingulate (green), medial occipital (yellow), supplementary motor area (orange), left posterior parietal (red), and right sensorimotor (cyan).

We grouped the 178 total dipoles into 11 clusters. We retained 8 clusters containing dipoles from more than half (>9) the total subjects (Fig 2): anterior parietal (12 subjects, 17 dipoles), left sensorimotor (11 subjects, 17 dipoles), right frontal (11 subjects, 14 dipoles), anterior cingulate (15 subjects, 27 dipoles), medial occipital (11 subjects, 13 dipoles), supplementary motor area (14 subjects, 21 dipoles), left posterior parietal (12 subjects, 13 dipoles), and right sensorimotor (13 subjects, 16 dipoles). We epoched the data from -300 to 800 ms around the auditory tone presentation, subtracted average activity across each epoch, and rejected epochs with amplitude outside ±75 μV to remove excessive artifact. We removed 1±1 trials for unaltered view low and 2±3 for virtual reality low, resulting in 102±4 trials for unaltered view low and 102±10 trials for virtual reality low (mean±SD). We only analyzed auditory events occurring while subjects were on the beam. We then calculated event-related potential activity time-locked to the auditory stimulus onset for each cluster. Auditory tone onset was set at time 0. We subtracted out 300 ms of average activity preceding the stimulus as baseline activity.

### 2.7. Statistical analyses

For main experiment mean physiological and behavioral data, we used non-parametric Friedman and Wilcoxon signed-rank tests due to non-normal data. We were not interested in comparing unaltered view low and virtual reality high due to difficulty interpreting any differences between these two conditions. We used Bonferroni correction for multiple comparisons, except for electrodermal activity, failures per minute, heart rate, and response time because these were planned, a priori comparisons. For the auxiliary experiment and fatigue comparison of percent change, differences across conditions were determined using non-parametric Friedman and post-hoc Wilcoxon signed-rank tests with Bonferroni correction for multiple comparisons. Non-EEG statistical analyses were performed using SPSS Statistics 22 (IBM SPSS Statistics 22.0, Armonk, NY, United States). For EEG event-related activity, we used EEGLAB permutation statistics with 8000 permutations to calculate pairwise comparisons and false discovery rate to correct for multiple comparisons [68]. Significance was determined to be less than 0.05 for all statistical tests.

## 3. Results

### 3.1. Survey results

Subjects did not experience substantial motion sickness from participating in the study. Results from the motion sickness survey are shown in Table 1, using a normalized percentage scale (0–100%). 0% suggests motion sickness was completely absent during testing, while 100% indicates motion sickness was fully present across all subjects. Questions are also grouped into subsections referencing different factors of motion sickness. All subsection scores were less than 25% across subjects, with a score of 5.8% for feeling nauseous. We were primarily concerned with adverse effects from nausea and dizziness, but subjects reported minimal effects from these areas. Also, subjects scored 15.1%±14.9% (mean±SD) on the Heights Interpretation Questionnaire, with 0% indicating no high heights apprehension.

**Table 1:** Motion sickness assessment results.

Feeling nauseous	5.8% (7.5%)
Feeling dizzy	12.0% (9.8%)
Feeling sweaty	21.3% (22.0%)
Feeling tired	20.2% (14.9%)
Total	14.2% (10.3%)

Mean motion sickness assessment scores are shown normalized from 0–100%, with standard deviation in parentheses (n = 19). The motion sickness assessment contains four subsections to analyze four factors of motion sickness: gastrointestinal (feeling nauseous), central (feeling dizzy), peripheral (feeling sweaty), and sopite (feeling tired).

### 3.2. Physiological and behavioral measures

We found significant increases in heart rate variability and heart rate frequency power in virtual reality high compared to virtual reality low (p = 0.015 and p = 0.006, respectively), as shown in Table 2. Both heart rate variability and heart rate frequency power did not significantly differ between unaltered view low and virtual reality low (p = 1.0 and p = 0.108). Electrodermal activity did not significantly differ between virtual reality conditions (p = 0.738), but did significantly increase in virtual reality low compared to unaltered view low (p = 0.009).

**Table 2:** Behavioral and physiological measures.

	Measure Value			P Value	
Measure	UVL	VRL	VRH	UVL|VRL	VRL|VRH
Heart Rate Variability (beats/min)	6.6 (1.5)	7.0 (1.6)	8.3 (2.4)	1.00	0.02*
Heart Rate Frequency Power (%)	45.6 (12.5)	51.8 (15.2)	58.9 (14.0)	0.11	<0.01*
Electrodermal Activity (counts/min)	8.2 (11.7)	15.1 (14.7)	13.7 (11.0)	<0.01*	0.74
Failures per Minute (counts/min)	7.1 (2.5)	26.1 (4.9)	24.8 (5.6)	<0.01*	1.00
Heart Rate (beats/min)	92.0 (7.9)	97.0 (8.7)	97.1 (10.6)	<0.01*	1.00
Response Time (s)	0.76 (0.25)	0.88 (0.25)	0.94 (0.27)	0.02*	0.10
Number of Beam Passes	43.0 (15.7)	26.6 (9.4)	24.7 (7.6)	<0.01*	0.38
Estimated Gait Speed (m/s)	0.18 (0.07)	0.13 (0.04)	0.13 (0.04)	0.03*	1.00

Mean behavioral and physiological measures are shown for unaltered view low (UVL), virtual reality low (VRL) and virtual reality high (VRH), with standard deviation in parentheses (n = 19 for each condition). The first 3 measures assessed stress induction. The other measures assessed cognitive and physical performance. All measures shown had significant Friedman test results across conditions. Pairwise comparison p-values are shown:

* denotes significant differences (p<0.05).

We only made two comparisons: 1) unaltered view low vs. virtual reality low and 2) virtual reality low vs. virtual reality high.

Subjects’ heart rate and response time significantly increased in virtual reality low compared to unaltered view low (p<0.001 and p = 0.018, respectively), indicating increased physical and cognitive exertion in virtual reality. We found no significant differences in heart rate (p = 1.0) and response time (p = 0.103) between virtual reality conditions.

Subjects’ balance performance significantly worsened in virtual reality low compared to unaltered view low (Table 2), as measured by failures per minute (p<0.001). There was no significant difference between virtual reality conditions (p = 1.0). In addition, subjects performed significantly more beam passes in unaltered view low compared to virtual reality low (p<0.001), likely because fewer step-offs occurred. We found no significant differences in beam passes between virtual reality conditions (p = 0.378). Subjects also beam-walked significantly faster in unaltered view low compared to virtual reality low (p = 0.027). We found no significant difference in gait speed between virtual reality conditions (p = 1.0). Gait speeds for all groups were lower than our desired speed of 0.22 m/s, potentially due to the difficulty of the beam-walking task.

### 3.3. Auxiliary experiment results

Our auxiliary experiment found few significant differences, and none of these differences appear to occur from wearing the virtual reality headset (Table 3). We only found significant effects for heart rate and heart rate variability (p<0.001 and p = 0.006). Heart rate increased when standing with the headset on and off compared to sitting with the headset on (p = 0.004 and p<0.001) and sitting with the headset off (p = 0.022 and p = 0.007). Comparisons within standing conditions and within sitting conditions had non-significant p-values (p = 1.0), suggesting that heart rate significantly changes due to alterations in physical task performance (sitting vs. standing), not from wearing the headset. While heart rate variability significantly differed across conditions, we did not find any significant pairwise comparisons. Sitting with the headset on decreased heart rate variability compared to standing with the headset off, but was not significant (p = 0.061). This difference may be caused by sitting vs. standing and has the opposite trend compared to the main experimental results. Electrodermal activity, response time, and heart rate frequency power did not significantly differ across conditions. Our auxiliary study of headset effects indicates that the significant differences found during beam-walking are likely not caused by just wearing the headset.

**Table 3:** Sitting/standing experiment results.

Measure	Sit Headset Off	Sit Headset On	Stand Headset Off	Stand Headset On
Heart Rate Variability (beats/min)	4.9 (1.5)	4.5 (1.7)	6.0 (1.5)	5.6 (2.0)
Heart Rate Frequency Power (%)	60.2 (15.5)	59.7 (15.9)	54.6 (16.5)	50.8 (14.4)
Electrodermal Activity (counts/min)	4.5 (9.9)	2.8 (5.3)	8.1 (12.7)	6.9 (10.1)
Heart Rate (beats/min)	72.7 (10.5)*	70.7 (10.2)*	88.4 (14.5)^✝^	86.0 (13.1)^✝^
Response Time (s)	0.63 (0.14)	0.66 (0.14)	0.62 (0.11)	0.66 (0.13)

* significantly different from standing conditions

^✝^ significantly different from sitting conditions

Mean physiological results are shown for the auxiliary headset experiment, with standard deviation in parentheses (n = 20). Pairwise significance was determined following a significant Friedman test (p<0.05). Heart rate and heart rate variability had significant Friedman test results, with significant pairwise differences in heart rate found between sitting and standing. No other significant differences were found.

### 3.4. Fatigue assessment results

**Fig 3:**
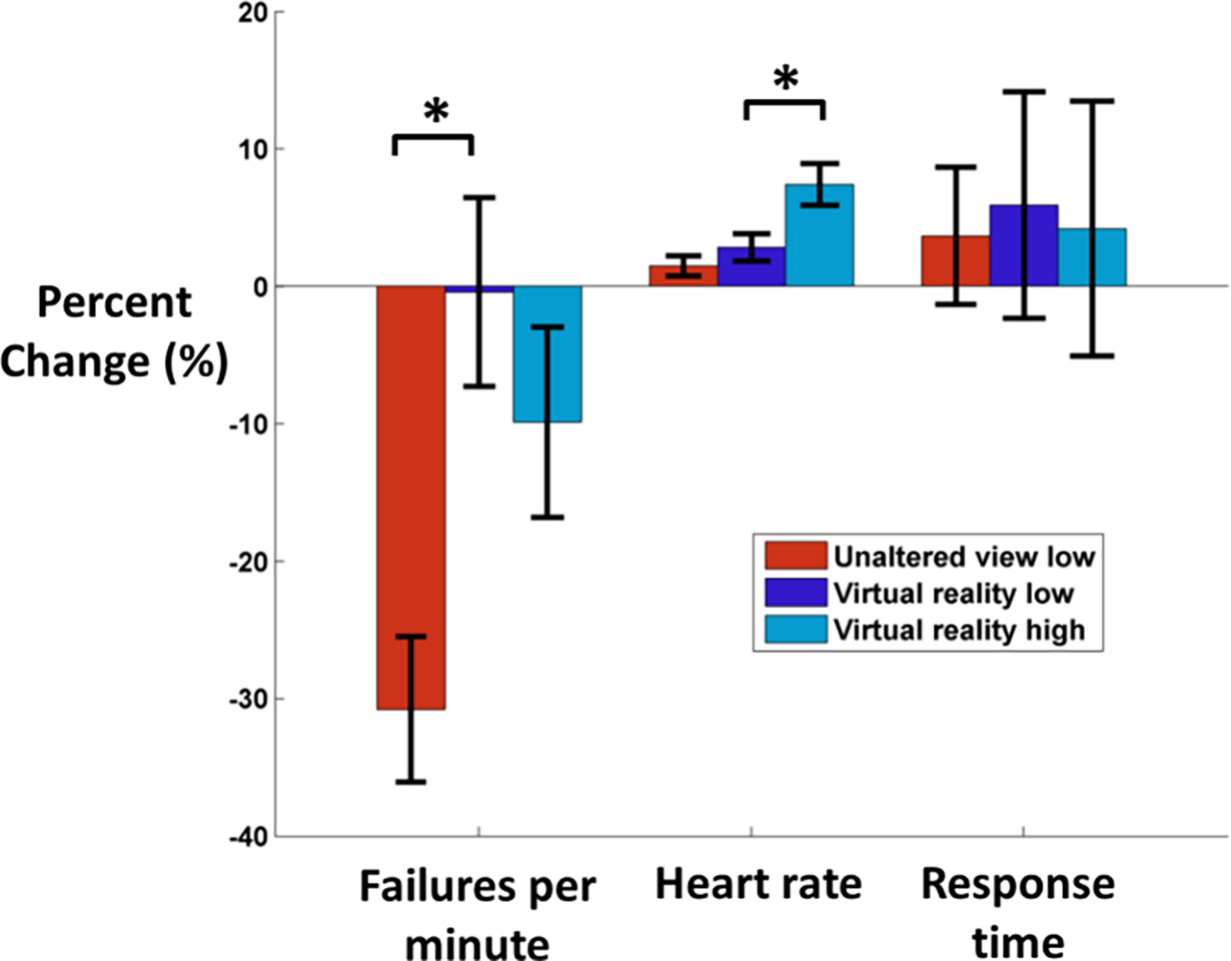
Percent change in failures per minute, heart rate, and response time. To assess fatigue effects, we calculated the percent change (mean±SE) between the first and last 3 minutes of each condition (n = 19). Failures per minute, heart rate, and response time are shown for unaltered view low (red), virtual reality low (dark blue), and virtual reality high (light blue). Negative percent change indicates that the value in the final 3 minutes decreased compared to the first 3 minutes. Failures per minute percent change significantly decreased in unaltered view low compared to virtual reality low. Heart rate percent change significantly increased in virtual reality high compared to virtual reality low. No other comparisons were significant between 1) unaltered view low vs. virtual reality low and 2) virtual reality low vs. virtual reality high.

We found significant differences across conditions related to fatigue for heart rate and failures per minute, but not for response time (Fig 3). Subjects’ percent change in failures per minute significantly increased in virtual reality compared to unaltered view low (p = 0.004), suggesting that virtual reality impaired motor acquisition. Percent change in failures per minute did not significantly differ between virtual reality conditions (p = 0.703). Heart rate percent change did not significantly differ between virtual reality low and unaltered view low (p = 0.489). Heart rate percent change significantly increased in virtual reality high compared to virtual reality low (p = 0.021), which may have been induced by stress from virtual high heights. Response time percent change did not significantly differ between unaltered view low and virtual reality low (1.0) and between virtual reality conditions (1.0), suggesting that subjects did not experience significantly different cognitive fatigue across conditions.

### 3.5. EEG data

**Fig 4:**
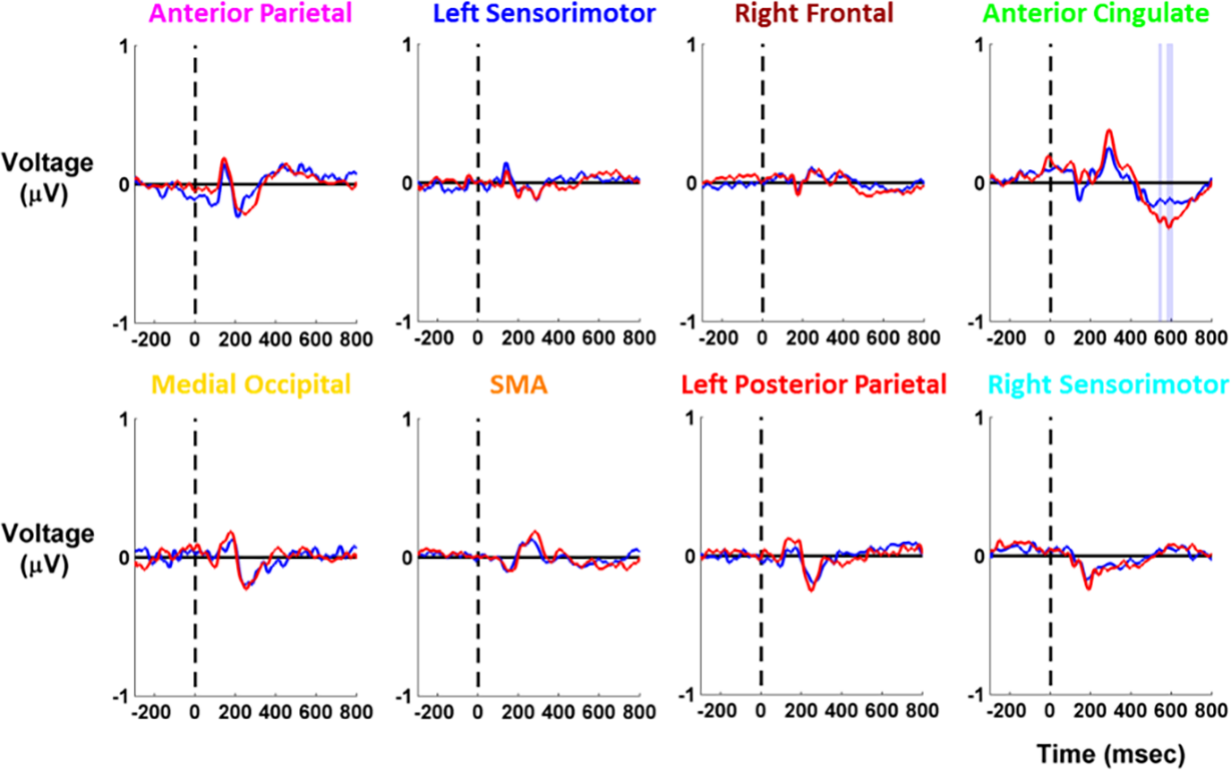
EEG event-related activity for cortical clusters. EEG event-related activity is shown for each cortical cluster (n = 19), with unaltered view low in red and virtual reality low in blue. Tone presentation occurred at 0 ms, preceded by 300 ms of baseline activity. We analyzed 800 ms following the tone presentation. Shading reflects the condition with significantly higher amplitude (red for unaltered view low, blue for virtual reality low). We found significant differences in the anterior cingulate cluster only.

We found significant differences in EEG event-related activity following the tone for the anterior cingulate cluster only (Fig 4). Because response time significantly differed between unaltered view low and virtual reality high but not between virtual reality conditions, we only compared EEG activity between unaltered view low and virtual reality low. In the anterior cingulate, virtual reality low peak activity significantly increased from 500–600 ms after the tone compared to unaltered view low. We were not concerned about motion artifact in the EEG recordings due to time-locking to a cognitive event and the slow gait speeds during the task (Table 2).

## 4. Discussion

We found that heart rate variability indicated increased stress at virtual high heights, which agreed with our first hypothesis. Subjects’ response time also significantly increased in virtual reality low compared to unaltered view low, but this difference was not due to fatigue. Significantly decreased EEG event-related peak activity in anterior parietal and anterior cingulate areas further corroborated our response time findings, confirming our second hypothesis.

We also found increased heart rate variability during virtual high heights exposure compared to low virtual heights exposure (Table 2). Increased heart rate variability during stress runs contrary to some studies [28,29], but agrees with a recent study using an acute stressor [30]. Such a discrepancy may arise from the paradigm that induced stress. In addition, a faster heart rate makes it more difficult to have high heart rate variability because there would be less time in a heartbeat cycle for variation in timing [69]. Heart rate variability significantly increased in virtual reality high compared to virtual reality low, despite both conditions having similar heart rates.

Another measure of stress, heart rate frequency power, was also greater during virtual high heights exposure compared with virtual low heights exposure Table 2). Heart rate frequency power focused on the percent of total power at all frequencies except 0.04–0.15 Hz. This excluded band contains a mix of sympathetic and parasympathetic responses [27]. The frequency power in this band decreased with high heights exposure, despite an increased heart rate in virtual reality. This suggests that the 0.04–0.15 Hz frequency band primarily measured parasympathetic response during the task, which significantly decreased with virtual high heights exposure. It is worth noting that parasympathetic changes can occur rapidly (milliseconds) compared to the sympathetic response (seconds) [70]. The sympathetic response may have been masked by increased physical exertion. While many studies focus on the high frequency power band (0.15–0.4 Hz) or on the ratio of low to high frequency power [71,72], a decrease in low frequency power with stress has also been documented [28].

While both heart rate variability metrics supported our hypothesis that stress was induced during virtual high heights exposure, electrodermal activity was primarily affected by physical exertion, instead of stress (Table 2). Electrodermal activity only measures the sympathetic response and has been shown to increase under both stress and physical loading [73]. Our findings contrast with stationary studies that have found decreased electrodermal activity in virtual reality [24,74]. This suggests that physical exertion primarily affected electrodermal activity. This is an important consideration for future experiments and highlights the challenges of quantifying stress, particularly during paradigms with high physical exertion. While we presented the phasic results of electrodermal activity here, we found similar results for the slower tonic component as well.

Subjects performed worse on the beam-walking task in virtual reality, based on failures per minute (Table 2). In addition, we found that subjects significantly lowered their failures per minute in unaltered view low viewing compared to virtual reality low (Fig 3). This indicates that both motor performance and motor acquisition were impaired by virtual reality use. Virtual reality use has been shown to worsen balance performance, with comparable stability to being blindfolded [75,76]. Subjects may have had difficulty adapting to virtual reality, reflected by increased physical and cognitive exertion.

The cognitive load of the subjects was greater during virtual reality use than during the unaltered view low condition, as measured by significantly increased response time in virtual reality low (Table 2). Significantly decreased EEG peak amplitude also indicated increased cognitive loading in the anterior cingulate cluster during virtual reality use (Fig 4). Similar decreases in event-related activity have been seen for this type of secondary auditory task when subjects performed a more challenging cognitive task [77]. The anterior cingulate is important for maintaining balance [40] as it is thought to perform error-detection [78][79]. Bogost et al. also found that the activity of the anterior cingulate and somatosensory area weakened during a reactive balance task when performing a challenging secondary task [80]. Dual-task interference during balance also reduces activity in sensorimotor and sensory areas in parietal cortex [81]. Other studies have found strong EEG activity in these regions during balance control with eyes open [40,82] and eyes closed [83]. Increased cognitive loading in the anterior cingulate may affect error detection while balancing, which may help explain why balance performance significantly worsened during virtual reality viewing.

While virtual reality induces realistic stress during virtual high height exposure, virtual reality headsets leave something to be desired during postural control. Low latency and limited field of view may have affected balance performance. The latency of the headset was 60 frames per second, but the movement generated by the Kinect was approximately 30 frames per second, which was likely noticeable to the user. In addition, the Kinect may not have provided ideal body tracking, which could break a subject’s sense of presence in virtual reality. Such breaks in presence can alter cognitive processing [84] and may have affected how realistic the virtual reality high heights experience felt to each subject. The headset also had a 110 degree field of view. In contrast, humans have at least a 180 degree field of view [85], and peripheral vision plays a primary role in worsen postural control [86,87]. However, virtual reality can still impair stability even when controlling for latency and field of view [88]. Virtual reality headsets continue to improve, and other options such as augmented reality may improve balance without the limitations of virtual reality headsets. This experiment establishes useful measures for assessing future virtual reality headsets in a dynamic setting.

## 5. Conclusions

Dynamic virtual reality exposure to high heights induces stress, indicating that this setup could provide realistic scenarios during dynamic balance training. However, technological limitations of virtual reality headsets currently limit the efficacy of balancing with a virtual reality headset. Balance performance, physical exertion, and cognitive loading provided a comprehensive quantification of how virtual reality use affects healthy young adults. Virtual reality technology needs to facilitate comparable balance to real world use before assisting patients with a fear of falling. Virtual reality technology will continue to develop, and we expect that future virtual reality headsets will achieve comparable results to balancing without a headset on.
